# Preliminary Psychometric Evaluation of the Romanian Version of the Hospital Anxiety and Depression Scale in Relapsing-Remitting Multiple Sclerosis

**DOI:** 10.3390/jcm15114165

**Published:** 2026-05-28

**Authors:** Lilia Böckels, Vitalie Lisnic, Daniel Alexa, Anna Belenciuc, Raul Andrei Crețu, Doina Ropot, Robert Valentin Bîlcu, Dan Iulian Cuciureanu

**Affiliations:** 1 Neurology Department, Rehabilitation Hospital, 700661 Iași, Romania; c.raulandy@yahoo.ro; 2 Faculty of Medicine, University of Medicine and Pharmacy “Grigore T. Popa”, 16 Universitatii Street, 700115 Iași, Romania; 3 Department of Neurology, State University of Medicine and Pharmacy “Nicolae Testemitanu”, MD-2004 Chisinau, Moldova; vitalie.lisnic@usmf.md (V.L.); belenciuc@gmail.com (A.B.); ropotdoina5@gmail.com (D.R.); 4 Doctoral School, University of Medicine and Pharmacy “Grigore T. Popa”, 700454 Iași, Romania; valentin.bilcu@d.umfiasi.ro; 5 Neurology Department, Faculty of Medicine, University of Medicine and Pharmacy “Grigore T. Popa”, 16 Universitatii Street, 700115 Iași, Romania; cuciureanudan@yahoo.com; 6 Neurology Department I, “Prof. Dr. N. Oblu” Emergency Clinical Hospital, 2 Ateneului Street, 700309 Iași, Romania

**Keywords:** multiple sclerosis, anxiety, depression, hospital anxiety and depression scale, preliminary psychometric evaluation, EDSS

## Abstract

**Background/Objectives:** Relapsing-remitting multiple sclerosis (RRMS) is frequently associated with anxiety and depressive symptoms, which significantly impact quality of life and clinical outcomes. The Hospital Anxiety and Depression Scale (HADS) is widely used to screen for psychological distress in medical populations. To perform a preliminary psychometric evaluation of the Romanian version of HADS in patients with RRMS, focusing on internal consistency and construct-related associations. **Methods:** This cross-sectional study included 60 RRMS patients. Internal consistency was assessed using Cronbach’s alpha and corrected item–total correlations. Construct-related evidence was explored through correlations with Expanded Disability Status Scale (EDSS) scores and disease duration, as well as known-groups comparisons based on disability level. **Results:** HADS demonstrated acceptable internal consistency (HADS-A α = 0.834; HADS-D α = 0.767). Depressive symptoms showed a moderate association with neurological disability (ρ = 0.456, *p* < 0.001), whereas no significant association was observed between anxiety symptoms and EDSS. Patients with higher disability had significantly higher HADS-D scores (*p* = 0.001), while HADS-A scores did not differ between groups. **Conclusions:** The Romanian version of HADS provides preliminary evidence of acceptable internal consistency and construct-related validity in RRMS. These findings support its potential use as a screening tool for psychological distress while highlighting the need for larger studies including factor analysis and test–retest reliability.

## 1. Introduction

Relapsing-remitting multiple sclerosis (RRMS) is a chronic inflammatory disease of the central nervous system characterized by complex autoimmune mechanisms leading to demyelination, progressive neurodegeneration, and cumulative disability over time. In addition to its classical neurological manifestations, MS is frequently associated with psychiatric comorbidities, particularly anxiety and depression, which significantly contribute to the overall burden of the disease. The prevalence of depression in patients with MS is up to three times higher than in the general population, while anxiety disorders are reported in approximately 30-40% of patients, irrespective of clinical phenotype or disease duration [1,2,3].

Anxiety and depressive symptoms exert a substantial impact on the clinical course of MS, negatively affecting quality of life, cognitive functioning, fatigue perception, and patient-reported disability. Depression has been associated with increased symptom severity and a marked reduction in quality of life and is considered an independent risk factor for unfavorable long-term clinical outcomes [4,5]. In this context, the systematic assessment of mental health represents a key component of multidisciplinary MS management, both in routine clinical practice and in research settings.

The use of standardized psychological screening instruments is essential for the early identification of anxiety and depressive symptoms in patients with chronic somatic diseases. The Hospital Anxiety and Depression Scale (HADS) is one of the most widely used self-report questionnaires for this purpose and was specifically designed to assess anxiety and depressive symptomatology in non-psychiatric medical contexts. The scale was originally developed by Zigmond and Snaith in 1983 with the aim of providing a reliable screening tool for emotional distress in patients with physical illnesses while minimizing the influence of somatic symptoms that could overlap with medical conditions. A major advantage of HADS is the exclusion of somatic items that could be confounded with symptoms of the underlying medical condition. This feature is particularly relevant in MS, where fatigue, sleep disturbances, and autonomic symptoms are common and may interfere with the accurate evaluation of emotional distress [6,7].

HADS consists of 14 items divided into two distinct subscales: anxiety (HADS-A) and depression (HADS-D), each comprising seven items. The instrument has demonstrated robust psychometric properties, including good internal consistency and satisfactory construct validity, and has been validated in numerous languages and widely applied across a variety of chronic medical conditions, including MS. Several studies have confirmed the reliability and validity of translated versions of HADS across different cultural contexts, including European populations and cohorts of patients with multiple sclerosis [8,9,10].

The application of a psychometric instrument within a specific cultural and linguistic context requires a rigorous adaptation and validation process to ensure the semantic, conceptual, and psychometric equivalence of the translated version. Cultural differences may influence how patients perceive, express, and report emotional symptoms, potentially affecting the accuracy of assessment if the instrument is not appropriately validated in the target population. The literature emphasizes the importance of adhering to international guidelines for the cross-cultural adaptation and psychometric evaluation of health-related questionnaires [11,12].

In Romania, the estimated prevalence of multiple sclerosis is approximately 70–90 cases per 100,000 inhabitants, with a steadily increasing number of diagnosed patients in recent years. This epidemiological context highlights the need for standardized and culturally adapted tools for the assessment of psychological comorbidities in Romanian-speaking MS populations.

Data regarding the standardized assessment of psychological symptoms in the Romanian MS population are limited, and the use of psychometrically validated instruments in this context remains insufficiently documented. This gap may lead to the underdiagnosis of anxiety and depressive symptoms and to an incomplete understanding of the psychological impact of MS on affected individuals. Given the high prevalence of psychiatric comorbidities and their relevance to disease outcomes and health-related behaviors, the validation of screening instruments used in routine clinical practice is warranted [13]. To our knowledge, this is among the first studies to evaluate the preliminary psychometric properties of the Romanian version of HADS in patients with multiple sclerosis, contributing to the limited evidence from Eastern Europe.

The aim of the present study was to evaluate the preliminary psychometric properties of the Romanian version of the HADS by assessing internal consistency and construct validity in a cohort of patients with MS. A preliminary evaluation of this instrument may facilitate its use in clinical practice and medical research, contributing to a more comprehensive and standardized assessment of anxiety and depressive symptoms in this population.

## 2. Materials and Methods

### 2.1. Study Design

This study was designed as an observational, cross-sectional investigation aimed at providing a preliminary psychometric evaluation of the Romanian version of HADS in patients diagnosed with MS. The study was conducted at a single center, within the framework of routine clinical assessments.

### 2.2. Study Sample

A total of 60 adult patients diagnosed with relapsing-remitting multiple sclerosis (RRMS) according to the revised McDonald criteria were included in the study. Participants were consecutively recruited from patients undergoing regular neurological follow-up at the study center. The inclusion criteria comprised age ≥ 18 years, a confirmed diagnosis of RRMS, the ability to understand and complete self-report questionnaires, and the provision of written informed consent, while the exclusion criteria included a prior diagnosis of major psychiatric disorders (e.g., schizophrenia or bipolar disorder), the presence of severe cognitive impairment interfering with questionnaire completion, and an acute relapse or intravenous corticosteroid treatment within the previous 30 days.

The sample size was determined based on the feasibility of consecutive recruitment within a single-center RRMS cohort and on the exploratory nature of the study. Given the 14-item structure of HADS, the inclusion of 60 participants provided more than four participants per questionnaire item, which was considered acceptable for a preliminary assessment of internal consistency and hypothesis-based construct validity. The sample size was not intended to support comprehensive psychometric modeling, such as exploratory or confirmatory factor analysis.

### 2.3. Assessment Instrument

The HADS is a self-report questionnaire consisting of 14 items divided into two subscales: HADS-A and HADS-D, each containing seven items. Each item is rated on a four-point Likert scale (0–3), resulting in subscale scores ranging from 0 to 21. Higher scores indicate greater severity of anxiety or depressive symptoms. For clinical interpretation, established cut-off values were used: scores of 0–7 were considered normal, 8–10 borderline, and 11–21 indicative of clinically significant symptoms.

### 2.4. Translation and Linguistic Adaptation

The Romanian version of HADS was obtained through a standardized linguistic adaptation process. Forward translation from English into Romanian was performed independently by two bilingual translators with a medical background and experience in clinical terminology. A backward translation into English was subsequently carried out by an independent translator blinded to the original version. Discrepancies were reviewed and resolved by an expert panel consisting of neurologists and researchers with experience in multiple sclerosis and questionnaire validation in order to ensure semantic and conceptual equivalence of all items. The finalized version was used in the present study.

### 2.5. Data Collection Procedure

The HADS questionnaire was administered individually under standardized conditions during a routine clinical visit. Participants completed the questionnaire autonomously under the supervision of a member of the research team, who provided clarification when necessary without influencing responses. Information regarding place of residence (urban vs. rural) was obtained from official administrative data recorded in the patients’ medical files. Neurological disability was assessed using the Expanded Disability Status Scale (EDSS), performed by a neurologist experienced in EDSS evaluation during routine clinical examination.

### 2.6. Statistical Analysis

Statistical analysis was performed using IBM SPSS Statistics for Windows, version 26.0 (IBM Corp., Armonk, NY, USA).

Internal consistency of the HADS subscales (HADS-A and HADS-D) was evaluated using Cronbach’s alpha coefficient, with values ≥ 0.70 considered acceptable according to established methodological guidelines. Corrected item–total correlations were calculated as the correlation between each individual item and the total score of the remaining items within the corresponding subscale in order to assess the contribution of each item.

Given the moderate sample size (*n* = 60) relative to the number of questionnaire items, exploratory factor analysis was not performed and was reserved for future studies with larger samples. Known-groups validity was assessed by comparing HADS subscale scores between patients with lower disability (EDSS ≤ 3) and higher disability (EDSS > 3) using the Mann–Whitney U test. The distribution of continuous variables was assessed using the Shapiro–Wilk test and visual inspection of histograms and Q-Q plots. Normally distributed variables were reported as mean ± standard deviation with minimum and maximum values, whereas non-normally distributed variables were reported as median and interquartile range when appropriate. No adjustment for multiple comparisons was applied given the exploratory nature of the study.

## 3. Results

A total of 60 RRMS patients were included (Table 1). Mean age was 40.5 ± 11.1 years (19–58), and mean disease duration was 9.9 ± 6.6 years (3–22). Most participants were female (66.7%), and the cohort was equally distributed between urban and rural residence (50.0% each).

Mean EDSS score was 2.9 ± 1.1 (range: 1–5). EDSS scores were distributed as follows: 13.3% of patients had EDSS 1, 33.3% had EDSS 2, 26.7% had EDSS 3, 23.3% had EDSS 4, and 3.3% had EDSS 5, reflecting predominantly mild-to-moderate disability.

Anxiety and depressive symptoms were assessed using HADS. Mean HADS-A score was 6.9 ± 3.7 (0–14), mean HADS-D score was 6.2 ± 3.6 (0–17), and mean total HADS score was 13.1 ± 6.1 (3–30). While the total score was reported as an indicator of overall psychological distress, clinical interpretation was based on established HADS-A and HADS-D cut-offs. Overall, mean subscale scores were within the normal range according to established HADS cut-off values, although a subset of patients exhibited borderline or clinically significant symptoms. The internal consistency of HADS was assessed in RRMS patients. HADS-A (7 items) showed good reliability (Cronbach’s α = 0.834), while HADS-D (7 items) demonstrated acceptable internal consistency (α = 0.767). Corrected item–total correlations ranged from 0.475 to 0.671 for HADS-A and from 0.347 to 0.643 for HADS-D, indicating the adequate contribution of all items.

The removal of any individual item did not result in a meaningful improvement in Cronbach’s alpha. Overall, these results confirm satisfactory reliability of HADS for assessing anxiety and depressive symptoms in RRMS. According to established HADS cut-off values, 34 patients (56.7%) presented anxiety scores within the normal range, while 16 patients (26.7%) were classified as having borderline anxiety symptoms and 10 patients (16.7%) exhibited clinically significant anxiety. Regarding depressive symptoms, 36 patients (60.0%) scored within the normal range, whereas 20 patients (33.3%) showed borderline depressive symptoms and 4 patients (6.7%) reached the clinical threshold for depression (Table 2).

Overall, these results suggest that although most RRMS patients did not meet criteria for clinically relevant anxiety or depression, a substantial proportion demonstrated borderline or clinically significant psychological distress, with anxiety symptoms being more frequent than depressive symptoms in this cohort. Construct validity of HADS was examined using Spearman correlation analyses with clinical disability and disease duration. EDSS scores demonstrated a moderate and statistically significant association with HADS-D (rho = 0.456, *p* < 0.001), suggesting that greater neurological disability in RRMS patients is accompanied by an increased depressive symptom burden (Table 3). In contrast, no significant relationship was identified between EDSS and HADS-A (rho = 0.085, *p* = 0.520), indicating that no statistically significant association between anxiety symptoms and disability was observed in this sample.

Disease duration was moderately correlated with EDSS (rho = 0.352, *p* = 0.006), consistent with the progressive accumulation of disability over time. Furthermore, HADS-A and HADS-D scores were significantly correlated (rho = 0.355, *p* = 0.005), reflecting the expected overlap between anxiety and depressive symptomatology and supporting the construct coherence of the instrument within this RRMS cohort.

Anxiety scores did not differ significantly between patients with lower and higher disability (U = 338, *p* = 0.813). In contrast, depressive symptoms were significantly more pronounced among patients with greater disability (mean rank 43.25 vs. 25.86; U = 148, *p* = 0.001) (Table 4), supporting the discriminative validity of the HADS-D subscale in distinguishing between clinically distinct levels of neurological impairment in RRMS.

## 4. Discussion

The present study provides a preliminary evaluation of the psychometric performance of the Romanian version of HADS in a cohort of patients with RRMS. The findings provide preliminary evidence regarding the internal consistency and construct-related associations of the instrument and contribute novel data to the limited body of literature addressing standardized psychological screening tools in Eastern European MS populations.

### 4.1. Internal Consistency and Reliability

In our cohort, both HADS subscales demonstrated satisfactory internal consistency, with Cronbach’s alpha coefficients indicating good reliability for HADS-A and acceptable reliability for HADS-D. These findings are consistent with more recent psychometric studies conducted in MS populations, which report alpha values generally exceeding 0.75 for anxiety and ranging between 0.70 and 0.85 for depression across diverse cultural contexts [14,15]. Importantly, corrected item–total correlations in our study confirmed that all items contributed meaningfully to their respective subscales, supporting the internal coherence of the Romanian version.

Recent methodological literature emphasizes that reliability estimates in chronic neurological populations should be interpreted in the context of symptom heterogeneity and disease-related variability, particularly when psychological constructs are assessed using brief screening tools [16]. From this perspective, the reliability indices observed in our sample are comparable to those reported in larger multicenter studies and support the use of HADS as a screening rather than diagnostic instrument in MS care.

### 4.2. Symptom Distribution and Clinical Relevance

Although mean HADS-A and HADS-D scores in our cohort fell within the normal range, a considerable proportion of patients exhibited borderline or clinically relevant anxiety and depressive symptoms. This pattern aligns with contemporary evidence suggesting that subclinical psychological distress is highly prevalent in MS and may represent an intermediate stage between emotional well-being and overt psychiatric disorders [17]. Recent longitudinal data indicate that patients with borderline anxiety or depression scores are at increased risk of symptom progression and functional decline over time, particularly when psychological distress remains unrecognized and untreated [18].

Anxiety symptoms were more prevalent than depressive symptoms in our sample, a finding that mirrors observations from recent population-based MS studies and registry analyses. Anxiety in MS has been increasingly conceptualized as a response to disease unpredictability, fear of future disability, and uncertainty related to treatment outcomes, rather than a direct consequence of neurological impairment alone [19]. These considerations underscore the importance of routine anxiety screening even in patients with relatively preserved physical function.

### 4.3. Construct Validity and Relationship with Disability

Construct validity of the Romanian HADS was supported by significant associations between depressive symptoms and neurological disability, as assessed by EDSS. The moderate correlation observed between HADS-D and EDSS is consistent with recent evidence indicating that depression in MS is closely linked to accumulated disability, loss of autonomy, and reduced participation in daily activities [20,21]. Importantly, EDSS should be interpreted as an external clinical correlate rather than a criterion standard for validating HADS. Therefore, the observed associations provide hypothesis-based construct validity evidence rather than direct confirmation of the instrument’s diagnostic validity.

No significant association was observed between anxiety symptoms and EDSS in this sample. This finding may reflect differences in the underlying determinants of anxiety compared to depression, as well as the influence of psychosocial factors, coping strategies, and illness perceptions, which are not captured by neurological disability scales.

The observed correlation between HADS-A and HADS-D scores reflects the expected overlap between anxiety and depressive symptomatology while supporting their partial independence as measured by HADS. Contemporary psychometric analyses using bifactor and network models have further demonstrated that HADS captures both a general distress component and distinct anxiety- and depression-specific dimensions, reinforcing the conceptual validity of separate subscale interpretation in MS populations. The lack of significant group differences for anxiety does not diminish the clinical relevance of HADS-A. Instead, it highlights the complexity of anxiety in MS and the need for complementary assessment strategies that address psychosocial stressors, illness-related fears, and cognitive–emotional factors beyond neurological disability [22].

### 4.4. Implications for Clinical Practice and Research

From a clinical standpoint, our findings support the implementation of the Romanian version of HADS as a feasible and informative screening tool in routine MS care. Recent international recommendations emphasize the importance of systematic mental health screening in neurological clinics, particularly given the strong associations between psychological distress, treatment adherence, health behaviors, and patient-reported outcomes [18,21]. The brevity of HADS and its exclusion of somatic items represent significant advantages in MS, where fatigue and physical symptoms may confound mood assessment when using other instruments.

In research settings, the availability of a validated Romanian HADS facilitates inclusion of psychological variables in observational and interventional MS studies and enables cross-cultural comparisons with international cohorts. This is particularly relevant in light of emerging evidence that psychological distress may influence disease perception, self-management behaviors, and engagement with disease-modifying therapies [18].

### 4.5. Limitations

- The study included a relatively moderate sample size, which may limit the statistical power and generalizability of the results.
- The research was conducted in a single-center cohort; therefore, the findings may not fully represent the broader RRMS population.
- Due to the small number of participants in some EDSS categories, disability levels were dichotomized, reducing the ability to explore differences across the full EDSS spectrum.
- Exploratory or confirmatory factor analysis could not be reliably performed given the limited sample size for a 14-item questionnaire. Future studies with larger, multicenter cohorts are needed to examine the factorial structure of the Romanian HADS in RRMS.
- Test–retest reliability was not assessed because HADS was administered only once within the cross-sectional study design; therefore, the temporal stability of the Romanian HADS could not be evaluated.
- Criterion validity was not examined, as no external gold-standard psychiatric scales were available for comparison.
- The cross-sectional design prevents conclusions regarding causality or longitudinal changes in anxiety and depression over time.

## 5. Conclusions

This study provides preliminary evidence supporting the psychometric performance of the Romanian version of HADS in patients with RRMS. Both subscales demonstrated acceptable internal consistency, indicating reliable measurement of anxiety and depressive symptoms. Construct-related analyses suggested an association between depressive symptoms and neurological disability, while no statistically significant association was observed between anxiety symptoms and disability level.

These findings support the potential utility of HADS as a screening tool for psychological distress in RRMS populations. However, larger multicenter studies incorporating factorial validation, test–retest reliability, and longitudinal assessment are required to establish comprehensive psychometric validation.

## Figures and Tables

**Table 1:** General characteristics of the study sample for HADS (*n* = 60): HADS, Hospital Anxiety and Depression Scale; HADS-A, Hospital Anxiety and Depression Scale-Anxiety; HADS-D, Hospital Anxiety and Depression Scale-Depression; EDSS, Expanded Disability Status Scale; SD, standard deviation.

Age, years mean ± SD	40.5 ± 11.1 years (range: 19–58)
Disease duration (years), mean ± SD	9.9 ± 6.6 years (range: 3–22)
Sex *n* (%)	
Female: 40 (66.7%)	Male: 20 (33.3%)
Residence *n* (%)	
Urban = 30 (50%)	Rural = 30 (50%)
EDSS *n* (%)	
0	0 (0%)
1	8 (13.3%)
2	20 (33.3%)
3	16 (26.7%)
4	14 (23.3%)
5	2 (3.3%)
HADS	
HADS-A (Anxiety) mean ± SD	6.9 ± 3.7 (range: 0–14)
HADS-D (Depression) mean ± SD	6.2 ± 3.6 (range: 0–17)
HADS Total mean ± SD	13.1 ± 6.1 (range: 3–30)

**Table 2:** Severity categories of anxiety and depressive symptoms according to HADS: HADS, Hospital Anxiety and Depression Scale; HADS-A, Hospital Anxiety and Depression Scale-Anxiety; HADS-D, Hospital Anxiety and Depression Scale-Depression.

Category (Score)	HADS-A Anxiety *n* (%)	HADS-D Depression *n* (%)
Normal (0–7)	34 (56.7%)	36 (60%)
Borderline (8–10)	16 (26.7%)	20 (33.3%)
Clinical (11–21)	10 (16.7%)	4 (6.7%)

**Table 3:** Correlations between HADS subscales and clinical variables: HADS, Hospital Anxiety and Depression Scale; HADS-A, Hospital Anxiety and Depression Scale-Anxiety; HADS-D, Hospital Anxiety and Depression Scale-Depression; EDSS, Expanded Disability Status Scale.

Correlation	Spearman’s rho	*p*-Value
Disease duration—EDSS	0.352	0.006
EDSS—HADS-A (Anxiety)	0.085	0.520
EDSS—HADS-D (Depression)	0.456	<0.001
Disease duration—HADS-A	−0.235	0.071
Disease duration—HADS-D	−0.014	0.916
HADS-A—HADS-D	0.355	0.005

**Table 4:** Comparison of HADS subscales according to disability level: HADS, Hospital Anxiety and Depression Scale; HADS-A, Hospital Anxiety and Depression Scale-Anxiety; HADS-D, Hospital Anxiety and Depression Scale-Depression; EDSS, Expanded Disability Status Scale.

Subscale	EDSS ≤ 3 (N = 44) Mean Rank	EDSS > 3 (N = 16) Mean Rank	Mann–Whitney U	*p*-Value
HADS-A (Anxiety)	30.18	31.38	338	0.813
HADS-D (Depression)	25.86	43.25	148	0.001

## Data Availability

The original contributions presented in this study are included in the article. Further inquiries can be directed to the corresponding authors.

## Abbreviations

HADS	Hospital Anxiety and Depression Scale
HADS-A	Hospital Anxiety and Depression Scale-Anxiety
HADS-D	Hospital Anxiety and Depression Scale-Depression
RRMS	Relapsing-Remitting Multiple Sclerosis
MS	Multiple Sclerosis
EDSS	Expanded Disability Status Scale
SD	Standard Deviation
